# Effects of pitavastatin versus atorvastatin on the peripheral endothelial progenitor cells and vascular endothelial growth factor in high-risk patients: a pilot prospective, double-blind, randomized study

**DOI:** 10.1186/s12933-014-0111-1

**Published:** 2014-07-16

**Authors:** Liang-Yu Lin, Chin-Chou Huang, Jia-Shiong Chen, Tao-Cheng Wu, Hsin-Bang Leu, Po-Hsun Huang, Ting-Ting Chang, Shing-Jong Lin, Jaw-Wen Chen

**Affiliations:** 1 Institute of Pharmacology, National Yang-Ming University, Taipei, Taiwan; 2 Division of Endocrinology and Metabolism, Department of Medicine, Taipei Veterans General Hospital, Taipei, Taiwan; 3 Faculty of Medicine, National Yang-Ming University, Taipei, Taiwan; 4 Department of Education, Taipei Veterans General Hospital, Taipei, Taiwan; 5 Cardiovascular Research Center, National Yang-Ming University, Taipei, Taiwan; 6 Division of Cardiology, Department of Medicine, Taipei Veterans General Hospital, Taipei, Taiwan; 7 Department of Medicine, Healthcare and Management Center, Taipei Veterans General Hospital, Taipei, Taiwan; 8 Institute of Clinical Medicine, National Yang-Ming University, Taipei, Taiwan; 9 Department of Medical Research, Taipei Veterans General Hospital, Taipei, Taiwan

**Keywords:** Pitavastatin, Atorvastatin, Hypercholesterolemia, Endothelial progenitor cell, Endothelial nitric oxide synthase, Vascular endothelial growth factor

## Abstract

**Background:**

Circulating endothelial progenitor cells (EPCs) reflect endothelial repair capacity and may be a significant marker for the clinical outcomes of cardiovascular disease. While some high-dose statin treatments may improve endothelial function, it is not known whether different statins may have similar effects on EPCs.This study aimed to investigate the potential class effects of different statin treatment including pitavastatin and atorvastatin on circulating EPCs in clinical setting.

**Methods:**

A pilot prospective, double-blind, randomized study was conducted to evaluate the ordinary dose of pitavastatin (2 mg daily) or atorvastatin (10 mg daily) treatment for 12 weeks on circulating EPCs in patients with cardiovascular risk such as hypercholesterolemia and type 2 diabetes mellitus (T2DM). Additional in vitro study was conducted to clarify the direct effects of both statins on EPCs from the patients.

**Results:**

A total of 26 patients (19 with T2DM) completed the study. While the lipid-lowering effects were similar in both treatments, the counts of circulating CD34^+^KDR^+^EPCs were significantly increased (from 0.021 ± 0.015 to 0.054 ± 0.044% of gated mononuclear cells, *P* < 0.05) only by pitavastatin treatment. Besides, plasma asymmetric dimethylarginine level was reduced (from 0.68 ± 0.10 to 0.53 ± 0.12 μmol/L, *P* < 0.05) by atorvastatin, and plasma vascular endothelial growth factor (VEGF) level was increased (from 74.33 ± 32.26 to 98.65 ± 46.64 pg/mL, *P* < 0.05) by pitavastatin. In the *in vitro* study, while both statins increased endothelial nitric oxide synthase (eNOS) expression, only pitavastatin increased the phosphorylation of eNOS in EPCs. Pitavastatin but not atorvastatin ameliorated the adhesion ability of early EPCs and the migration and tube formation capacities of late EPCs.

**Conclusions:**

While both statins similarly reduced plasma lipids, only pitavastatin increased plasma VEGF level and circulating EPCs in high-risk patients, which is probably related to the differential pleiotropic effects of different statins.

**Trial registration:**

This trial is registered at ClinicalTrials.gov, NCT01386853.

## Introduction

Coronary heart disease (CHD) remains the leading cause of death globally [[1]]. Elevated low-density lipoprotein cholesterol (LDL-c) levels play a pivotal role in the pathogenesis of atherosclerosis and LDL-c–lowering therapy can reduce cardiovascular morbidity and mortality [[2]]. Statins are the most effective drugs for LDL-c lowering and exert a number of so-called “pleiotropic” vasculoprotective effects including production of nitric oxide (NO) by the upregulation of endothelial NO synthase (eNOS), improvement of endothelial function, and induction of the expression of angiogenic factors such as vascular endothelial growth factor (VEGF) [[3]].

Accumulating evidence indicates that clinical treatment of statins may not only markedly reduce plasma LDL-c levels but also significantly improve the clinical outcomes of the patients at high risk of atherosclerosis cardiovascular disease. It has been suggested that both the LDL-c lowering and the pleiotropic effects may contribute to the clinical benefits of statins [[4],[5]]. There have been several different statins as a class available for clinical use. Among them, atorvastatin, a potent lipophilic statin, is most widely used in the world. Though were shown with additional pleiotropic vasculoprotective effects, particularly in high dose, the potent statins such as atorvastatin may cause some unfavorable metabolic effects including the exacerbation of insulin resistance [[4],[6]]. On the other hand, pitavastatin, as the latest statin in the same class available for clinical use, is minimally metabolized by CYP2C9, which is different from other statins including simvastatin, lovastatin, fluvastatin and atorvastatin that are mainly metabolized by CYP 3A4 [[7]]. However, it was not known whether pitavastatin, with different drug metabolism, may have similar pleiotropic effects in term of direct vascular protection of other statin such as atorvastatin in clinical settings.

Endothelial progenitor cells (EPCs), as a specific group of cells generated from bone marrow, have been demonstrated to maintain endothelial integrity and to generate new blood vessels. It was further indicated that the levels of circulating EPCs, by various definitions, could reflect *in vivo* endothelial repair capacity [[8]]. In patients with increased cardiovascular risk especially hyperipidemia and type 2 diabetes (T2DM), the circulating EPC count, as a surrogate marker for vascular function, may be reduced and associated with the long-term clinical outcomes [[9]-[11]]. Recent clinical evidence suggest the effects of different statins on vascular endothelial function as well as EPCs may vary in different clinical settings [[3],[12]-[17]], which might be due to the different metabolic nature of the statins and/or the different natures of the study cohorts [[3]]. Besides, the statin treatment in previous studies was principally with a relatively high dose (usually 3 to 4 times of the standard dose) for a relatively short duration (usually for 4 weeks or less, only one for 8 weeks). The long-term clinical effects of statin treatment with an ordinary dose similar to that used in most daily practice were not known.

Given the potential variation in pleiotropic vascular protection effects of different statins in clinical settings, this study aimed to examine whether long-term treatment with an ordinary dose of two different statins, atorvastain and pitavastatin, may have different effects on circulating EPC number and function in a group of high-risk patients including those with hyperlipidemia and T2DM. Additional *in vitro* study was also conducted to elucidate the potential mechanisms of the direct effects of different statins on EPCs from the patients.

## Materials and methods

### Clinical study

#### Study population and design

We conducted a single-center, double-blinded, randomized clinical trial as a substudy of “Efficacy and safety of pitavastatin and atorvastatin: a 12-week, randomized, double-blinded, active controlled study in high risk patients with hyperlipidemia” (registered at ClinicalTrials.gov, NCT01386853). While the main protocol was conducted in several different hospitals, this study was a substudy supplemental to the main study that was only conducted in one of the hospitals. During a period of 3 months, a series of 34 consecutive patients were screened and enrolled in a 1:1 ratio to atorvastatin or pitavastatin treatment in a medical center in Taipei, Taiwan. The inclusion criteria included (1) Patient with fasting LDL-c > 100 mg/dL. (2) Patient with at least one of the following description: (i) CHD (ii) T2DM (iii) patients with more than 2 risk factors other than LDL-c. Subjects were excluded on the basis of the following criteria: (1) history of taking medication known to alter blood lipid profiles; (2) history of type 1 DM or receiving insulin/insulin analogs; (3) serum triglyceride level > 400 mg/dL; (4) history of cardiovascular disease diagnosed within 3 months; (5) renal and liver function impairment. The study objective is to compare pitavastatin 2 mg versus atorvastatin 10 mg given once daily for 12 weeks on the effect of lipid profile and the number of circulating EPCs. This study was approved by local institute research ethics committee (VGH IRB No: 2011-09-015IB). All participants gave written informed consents before entering the study.

#### Human EPC isolation and flow cytometry

The detailed protocol for celll isolation has been discribed in our previous studies [[18]]. In brief, a total of 20 cc of blood was drained from the peripheral veins of the upper arms in each patients in the morning hours after an overnight fasting. Total mononoculear cells (MNCs) were isolated from peripheral blood by density gradient centrifugation with Histopaque-1077 (1.077 g/ml; Sigma, St. Louis, MO, USA). The cells were dissociated with cell dissociation buffer and incubated with monoclonal antibodies against human KDR (R&D, Minneapolis, MN, USA) followed by Allophycocyanin-conjugated secondary antibody, with the peridinin chlorophyll protein(PerCP)-labeled monoclonal antibodies against human CD45 (BD Biosciences, San Jose, CA, USA), with the PE-conjugated monoclonal antibody against human CD133 (Miltenyi Biotec, Germany), and with FITC-conjugated monoclonal antibodies against human CD34 (BioLegend, San Diego, CA, USA) in the dark for 30 minutes. After incubation, cells were fixed in 2% paraformaldehyde and analyzed by flow cytometry performed using a FACS Vantage Flow Cytometer (BD Biosciences, San Jose, CA, USA). Each analysis included 150,000 events, after selection for CD45-positive cells and exclusion of debris. Gated CD34- or CD133-positive cells were subsequently examined for the expression of KDR.

#### Serum biomarkers

All of the blood samples were obtained after an overnight fasting. The vascular inflammation, atherosclerosis, and angiogenesis related biomarkers including high-sensitive C-reactive protein (hs-CRP), total nitric oxide, interleukin-10, tumor necrosis factor-α, stromal cell-derived factor-1α (SDF-1α), and VEGF were analyzed by Quantikine human ELISA kits (R&D systems, Minneapolis, MN, USA). The serum level of asymmetric dimethylarginine (ADMA) was also measured by human ELISA kit (DLD Diagnostika GmbH, Hamburg, Germany). All the measurements were conducted according to the standard methods in the operation mannul.

### In vitro study

#### Statins

The atorvastatin was purchased from Sigma-Aldrich (St. Luis, MO, U.S.A). The pitavastatin was kindly provided as a gift by Professor Kensuka Egashira in Fukuoka, Japan.

#### Cultivation for early and late EPCs

The detailed protocos of the culture for early and late (outgrowth) EPCs have been discribed in our previous studies [[18],[19]]. In brief, total mononoculear cells (MNCs) were isolated from peripheral blood in the study patients by density gradient centrifugation with Histopaque-1077 (1.077 g/ml; Sigma, St. Louis, MO, USA). The MNCs (5 × 10^6^) were plated in 2 ml endothelial growth medium (EGM-2 MV; Cambrex, East Rutherford, NJ, USA), with supplements on fibronectin-coated 6-well plates. Under daily observation, after 4 days of culturing, medium was changed and non-adherent cells were removed; attached “early EPCs” appeared to be elongated with a spindle shape. A certain number of early EPCs could continue to grow into colonies of “late (outgrowth) EPCs”, which emerged 2–4 weeks after the start of MNC culture. The characteristics of the early and the late EPCs have been well demonstrated previously [[18],[19]].

#### MTT (3-[4,5-dimethylthiazol-2-yl]-2,5-diphenyl tetrazolium bromide) assay

The MTT (3-[4,5-dimethylthiazol-2-yl]-2,5-diphenyl tetrazolium bromide) was performed to evaluate the mitochondrial activity and viability of either early or late EPCs from the patients with hypercholesterolemia and T2DM. After pre-treated with statins for 24 hours, 1 mg/mL of MTT (Sigma, St. Louis, MO, USA) was added to the EPC medium culture and incubated for an additional 3–4 hours. The medium was then removed and the cells were solubilized in isopropanol. The amount of the dye released from the cells was measured with a spectrophotometer at 570 nm and subtracted background at 690 nm. An increase in the number of viable cells results in an increase in the amount of MTT formed and, therefore, in absorbance.

#### Adhesion assay on early EPCs

The adhesion assay for early EPCs was determined to elucidate the effect of both statins (10^−7^ M) on the early EPCs. The adhesion assay was performed with the methods as described in detail previously [[18],[19]].

#### Proliferation assay on late EPCs

The proliferation assay was performed to elucidate the effect of both statins (10^−5^ to 10^−9^ M, for 24 hours) on late EPCs. The proliferation assay was performed with the methods as described in detail previously [[18],[19]].

#### Migration assay on late EPCs

The effect of statins on functional assays of late EPCs including migration assay and tube formation assay were examined after pre-treatment of different statins (10^−7^ M) for 24 hours. The migratory function of late EPCs was evaluated by a modified Boyden chamber assay (Transwell, Coster, San Diego, CA, USA) [[19]]. Briefly, isolated EPCs were detached as described above with trypsin/EDTA and then 1×10^4^ late EPCs were placed in the upper chambers of 24-well Transwell plates with polycarbonate membrane (8-mm pores) with serum-free endothelial growth medium; VEGF (50 ng/ml) in medium was placed in the lower chamber. After incubation for 24 hours, the membrane was washed briefly with PBS and fixed with 4% paraformaldehyde. The membrane was then stained using hematoxylin solution and carefully removed. The magnitude of migration of the late EPCs was evaluated by counting the migrated cells in six random high-power (100X) microscopic fields.

#### EPC tube formation assay on late EPCs

The EPC tube formation assay was performed using the In Vitro Angiogenesis Assay Kit (Chemicon). ECMatrix gel solution was thawed overnight at 4°C, mixed with ECMatrix diluent buffer, and placed in a 96-well plate for 1 h at 37°C to allow the matrix solution to solidify. Late EPCs were harvested with trypsin/EDTA, as described above, and 1X10^4^ EPCs were placed onto a matrix with EGM-2 MV medium and incubated at 37°C for 16 h. Tubule formation was inspected with an inverted light microscope (100X). Six representative fields were used to determine the average of the total area of complete tubes formed by cells using the computer software, Image-Pro Plus.

#### Western blot analysis

Early and late EPCs derived from the patients with T2DM were cultured on the on fibronectin-coated 6-well plates for examination of endothelial nitric oxide synthase (eNOS) protein expression determined by Western blotting after the statins exposure. The Western blot analyses were performed with the methods as described in detail previously [[19]].

#### Statistical analysis

Continuous variables were expressed as mean ± SDs, and categorical variables were presented as frequencies and percentages. Differences between two groups were analyzed using a two-tailed Student’s *t* test or one-way analysis of variance. Given the specific experiment purpose of this clinical study, the changes of the major index parameters were compared before and after the treatment within the group by the paired Student’s *t* test. All tests were two sided and a value of *P* < 0.05 was considered statistically significant. Statistical analysis was adequately performed with SPSS software (version 18; SPSS Inc., Chicago, Illinos).

## Results

### Clinical study

#### Baseline characteristics of the study patients

A series of 34 consecutive patients were initially enrolled in the out patient clinics of a national medical center in Taipei, Taiwan. Among them, 4 patients who did not meet the inclusion criteria, 2 patients who were with poor drugs compliances (missed more than 75% of test medications), 1 patient with early withdrawal (ALT more than 5 times of upper normal limits) and 1 patient died with choking in accident. They were excluded from the subsequent analyses. Finally, a total of 26 patients (11 males, aged 60 ± 9 years; 13 with pitavastatin and 13 with atorvastatin) completed the study. The baseline characteristics of the study patients (13 in each group) were presented in Table 1. There were no differences between the two treatment groups at baseline.

**Table 1:** Baseline characteristics of study subjects

	**Pitavastatin**	**Atorvastatin**	**P value**
	**(n = 13)**	**(n = 13)**	
Age (years)	60.1 ± 7.3	60.5 ± 10.3	0.90
Male, n (%)	6 (46.2%)	5 (38.5%)	0.69
Type 2 Diabetes Mellitus, n (%)	8 (61.5%)	11 (84.6%)	0.38
Hypertension, n (%)	10 (70.7%)	7 (53.8%)	0.67
Coronary artery disease, n (%)	2 (15.4%)	0 (0.0%)	0.14
Current smoker, n (%)	2 (15.4%)	1 (7.7%)	0.54
Waist Circumference (cm)	87.7 ± 6.3	91.7 ± 13.1	0.33
BMI (kg/m^2^)	25.2 ± 3.9	27.1 ± 3.8	0.22
SBP (mmHg)	118.2 ± 12.6	127.5 ± 19.6	0.17
DBP (mmHg)	76.2 ± 8.9	80.8 ± 11.1	0.26
HR (/min)	73.5 ± 11.7	75.7 ± 10.9	0.63
Creatinine (μmol/L)	76.9 ± 19.4	70.7 ± 17.7	0.25
ALT (U/L)	21.7 ± 13.5	33.6 ± 21.1	0.10
Glucose (mmo/L)	6.98 ± 1.39	6.28 ± 1.04	0.16
A1c (%)	6.8 ± 1.1	6.3 ± 0.5	0.18
Medication			
Metformin, n (%)	8 (61.5%)	11 (84.6%)	0.14
DPP-4 inhibitors, n (%)	2 (15.4%)	1 (7.7%)	0.54
PPAR-γ agonists, n (%)	0 (0%)	1 (7.7%)	0.31
ARB, n (%)	3 (23.1%)	4 (30.8%)	0.66
ACEI, n (%)	3 (23.1%)	2 (15.4%)	0.62
CCB, n (%)	5 (38.5%)	3 (23.1%)	0.40
Beta-blockers, n (%)	2 (15.4%)	2 (15.4%)	1.00
Diuretics, n (%)	1 (7.7%)	4 (30.8%)	0.14
Aspirin, n (%)	3 (23.1%)	1 (7.7%)	0.28

Values are mean ± standard deviation (SD) or number (%). ARB: angiotensin II receptor blockers; ACEI: angiotensin converting enzyme inhibitors; BMI: body mass index; CCB: calcium channel blockers; DBP: diastolic blood pressure; HR: heart beats ALT: analine amiontransferase; DPP-4 inhibitors: dipeptidyl peptidase-4 inhibitors; PPAR-γ agonists: peroxisome proliferator activator receptor-gamma agonists, SBP: systolic blood pressure.

#### Similar effects of the statin treatment on plasma lipid profiles and differential on glucose metabolism

The plasma lipid profiles including total cholesterol, LDL-c and apolipoprotein B levels were significantly and similarly decreased after 12-week treatment of pitavastatin or atorvastatin. While the fasting glucose level was not changed, the homeostasis model of assessment-insulin resistance (HOMA-IR) index was significantly increased (from 2.0 ± 1.0 to 3.9 ± 2.8, *p* = 0.002) after the 12-week treatment of atorvastatn. There were so much changes in pitavastatin group (Table 2).

**Table 2:** Effects of 4- and 12-week treatment of pitavastatin (PTV) and atorvastatin (ATV) on lipid profiles, systemic biomarkers and endothelial progenitor cells

	**Baseline**			**4 weeks**			**12 weeks**			**PTV#**	**ATV#**
	**PTV**	**ATV**	**P-value**	**PTV**	**ATV**	**P-value**	**PTV**	**ATV**	**P-value**	**P-value**	**P-value**
WBC (/cumm)	6223 ± 1790	7184 ± 1563	0.16	6784 ± 2106	7238 ± 1180	0.50	6338 ± 1809	6930 ± 1245	0.34	0.73	0.82
Glucose (mmol/L)	6.98 ± 1.39	6.28 ± 1.04	0.16	7.12 ± 1.71	6.54 ± 1.23	0.39	7.58 ± 2.54	6.54 ± 1.05	0.18	0.71	0.80
HOMA-IR	1.9 ± 1.2	2.0 ± 1.0	0.78				5.2 ± 4.8	3.9 ± 2.8	0.41	0.05	<0.05
hs-CRP (mg/dL)	0.18 ± 0.18	0.35 ± 0.37	0.16	0.17 ± 0.11	0.16 ± 0.19	0.90	0.20 ± 0.20	0.17 ± 0.20	0.88	0.93	0.17
TC (mmol/L)	5.20 ± 0.69	5.24 ± 0.64	0.89	3.73 ± 0.53	3.62 ± 0.36	0.53	3.65 ± 0.52	3.56 ± 0.37	0.58	<0.05	<0.05
TG (mmol/L)	1.53 ± 0.71	1.72 ± 0.59	0.46	1.25 ± 0.58	1.26 ± 0.28	0.94	1.15 ± 0.51	1.22 ± 0.39	0.72	0.26	<0.05
LDL-c (mmol/L)	3.70 ± 0.55	3.82 ± 0.85	0.68	2.12 ± 0.46	2.19 ± 0.45	0.68	2.14 ± 0.33	2.09 ± 0.51	0.76	<0.05	<0.05
HDL-c (mmol/L)	1.25 ± 0.25	1.18 ± 0.19	0.38	1.29 ± 0.33	1.18 ± 0.18	0.32	1.25 ± 0.28	1.19 ± 0.20	0.58	0.92	0.96
NOx (μmol/L)	70.8 ± 42.5	78.5 ± 96.8	0.80	75.3 ± 66.1	80.6 ± 62.9	0.85	79.5 ± 86.1	68.9 ± 46.4	0.71	0.95	0.91
IL-10 (pg/ml)	1.8 ± 1.2	1.5 ± 0.8	0.48	2.1 ± 1.6	2.1 ± 1.4	0.91	2.07 ± 1.6	1.7 ± 1.8	0.57	0.87	0.55
TNF-α (pg/ml)	3.6 ± 1.7	4.0 ± 2.8	0.67	3.3 ± 1.8	4.5 ± 2.8	0.22	2.8 ± 0.8	4.3 ± 3.2	0.12	0.45	0.91
ADMA (μmol/L)	0.61 ± 0.10	0.68 ± 0.10	0.09				0.56 ± 0.12	0.53 ± 0.12	0.55	0.25	<0.05
SDF-1α (pg/ml)	2619.9 ± 640.5	2851.4 ± 669.1	0.38	2673.5 ± 774.6	3043.3 ± 733.5	0.24	2603.6 ± 757.1	3085.5 ± 701.1	0.16	0.98	0.668
VEGF (pg/ml)	74.33 ± 32.26	69.86 ± 31.53	0.72	118.36 ± 50.05	67.65 ± 42.85	0.01	98.65 ± 46.64	74.04 ± 29.33	0.12	<0.05	0.898
EPC level (%)											
CD34^+^KDR^+^	0.021 ± 0.015	0.039 ± 0.039	0.16	0.035 ± 0.031	0.029 ± 0.024	0.59	0.054 ± 0.044	0.043 ± 0.029	0.45	<0.05	0.57

Values are mean ± standard deviation. #: Difference between baseline, 4 weeks and 12 weeks of treatment in the same group. WBC: white blood cell; HOMA-IR: Homeostasis model of assessment-insulin resistance; TC: total cholesterol; TG: Triglyceride; LDL-c: low-density lipoprotein cholesterol; HDL-c: high-density lipoprotein cholesterol; hs-CRP: high sensitivity C-reactive protein; NOx: total nitric oxide; IL-10: interleukin-10; TNF-α: tumor necrosis factor-α; ADMA: asymmetric dimethylarginine; SDF-1α: stromal cell-derived factor-1α; VEGF: vascular endothelial growth factor; EPC: endothelial progenitor cell.

#### Differential effects of the statin treatment on serum biomarkers

There were no significant changes in systemic biomarkers including serum hs-CRP, total NO, interleukin-10, TNF-α, and SDF-1α over time in both groups. However, atorvastatin treatment signifcantly reduced serum ADMA concentration (from 0.68 ± 0.10 to 0.53 ± 0.12, *p* = 0.009), which was not seen by pitavastatin treatment. On the other hand, the VEGF levels was significantly increased by the treatment of pitavastatin (from 74.33 ± 32.26 to 98.65 ± 46.64 pg/mL, *P* < 0.05), which was not seen by atorvastatin treatment (Table 2).

#### Differential effects of the statin treatment on circulating EPCs

**Figure 1:**
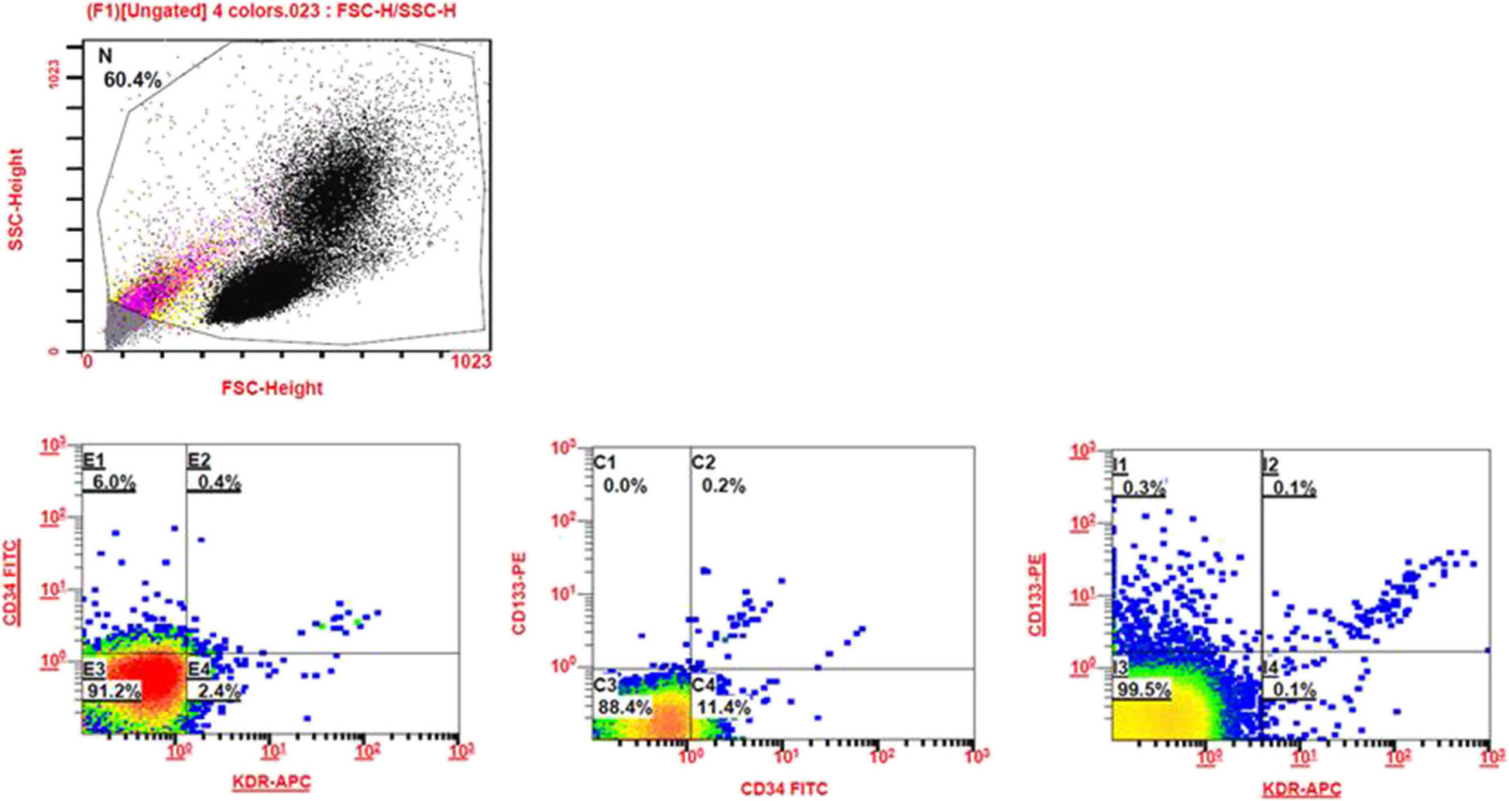
Representative flow cytometry analysis for quantifying the number of circulating endothelial progenitor cells (EPCs).

**Figure 2:**
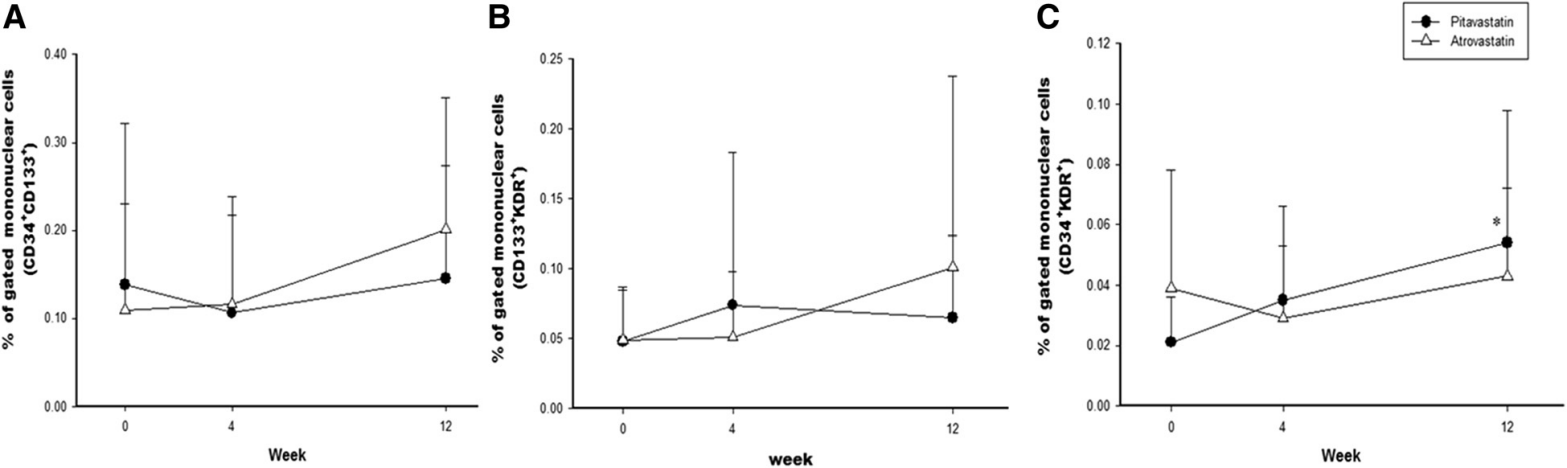
**Percentage of stained dual-positive endothelial progenitor cells (EPCs) of gated mononuclear cells analyzed by flow cytometry.** There are no differences of **(A)** CD34^+^CD133^+^ EPCs and **(B)** CD133^+^KDR^+^ EPCs between pitavastatin and atorvastatin groups. **(C)** The levels of circulating CD34^+^KDR^+^ EPCs are significantly increased after 12 week treatment with pitavastatin. *: *p* < 0.05 compared with baseline.

The peripheral blood MNCs were gated and granulocytes by the flow cytometry analysis and the gated MNCs with dual stained positive cells were shown in the Figure 1. There were no differences in the number of CD34^+^KDR^+^, CD34^+^CD133^+^ or CD133^+^KDR^+^ EPC-like cells between the two treatment groups at baseline (Table 2, Figure 2). Pitavastatin treatment for 12 weeks significantly increased the number of circulating EPCs (CD34^+^KDR^+^) (0.054 ± 0.044 after treatment v.s. 0.021 ± 0.015% of gated mononuclear cells at baseline, *p* < 0.05). There were no significant changes of CD34^+^KDR^+^ EPCs with atorvastatin treatment (Table 2, Figure 2).

#### The potential effects of the changes of serum lipid profiles and biomarkers on the changes of EPCs by pitavastatin treatment

Further analyses showed that the changes of apolipoprotein (Apo) A1 after 12-week treatment were insignificant in both statin groups (Pitavastatin: 133.8 ± 15.7 mg/dL to 135.5 ± 14.6 mg/dL, *p =* 0.54; Atorvastitan: 134.5 ± 19.8 mg/dL to 132.5 ± 16.2 mg/dL, *p =* 0.50) and no significant differences between two groups were noted before and after treatment (*p =* 0.93 at baseline; *p =* 0.63 at 12-week). Then, we analyzed the potential association between the differences of high-density lipoprotein cholesterol (HDL-c) or Apo A1 and that of CD34^+^/KDR^+^ EPCs or other biomarkers including hs-CRP, NOx, IL-10, TNF-α, ADMA, SDF-1α and VEGF after 12-week pitavastatin treatment. There were no any associations of the changes of HDL-c or Apo A1 with the changes of CD34^+^/KDR^+^ EPC number in patients with pitavastatin treatment. Therefore, the changes of serum HDL-c or Apo A1 may be not related to the increment of EPCs (CD34^+^/KDR^+^) number by pitavastatin treatment in this study.

### In vitro study

**Figure 3:**
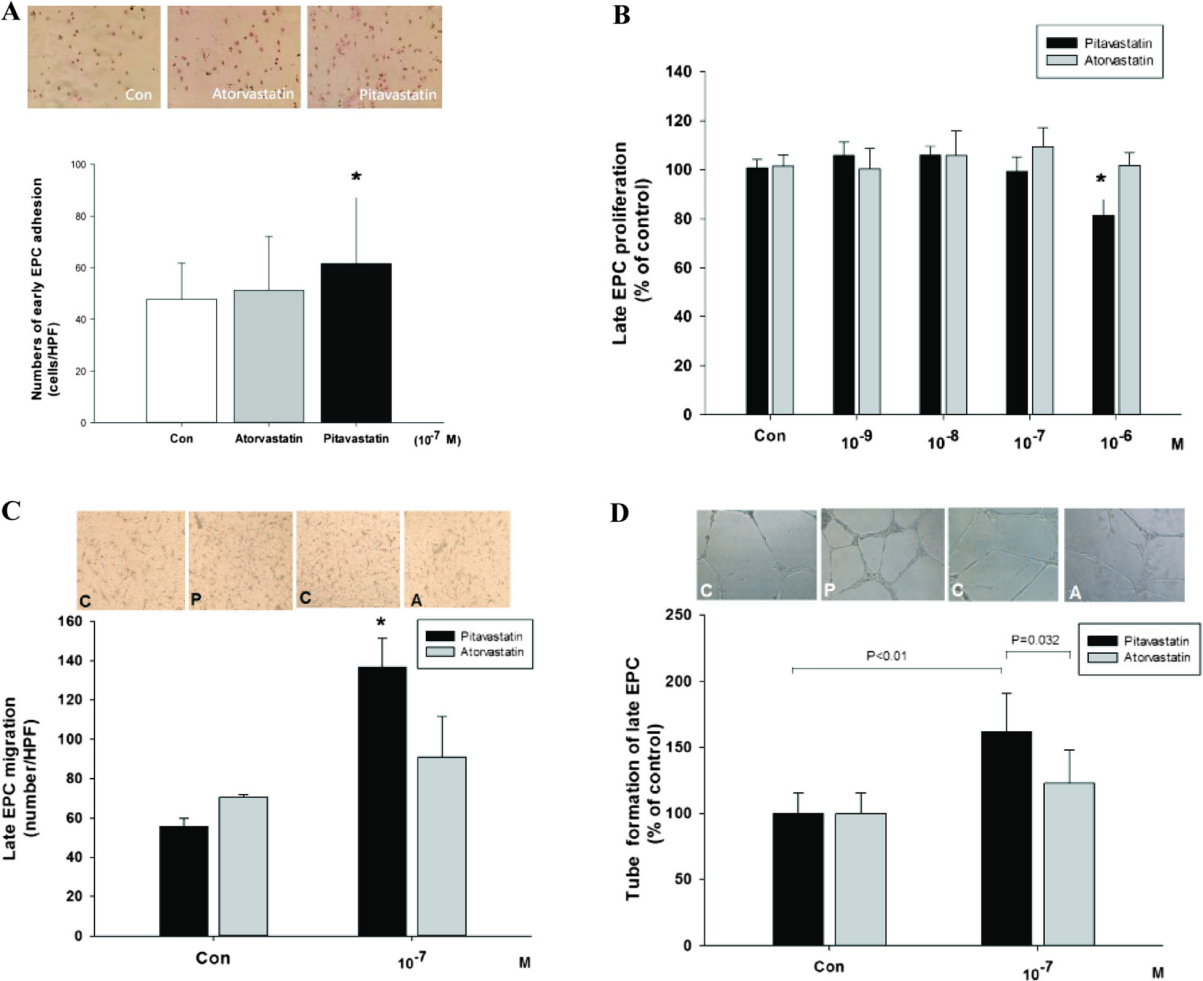
**Pitavastatin significantly increased the adhesion function of early endothelial progenitor cells (EPCs).** Early EPCs were pretreated with pitavastatin and atorvastatin (10^−7^ M) for 24 hours. The number of adhesion of early EPC was analyzed by fibronectin-adhesion assay (n = 12) **(A)**. The effects of pitavastatin and atorvastatin on the proliferation of late EPCs, analyzed by MTT assay after treatment with different doses of statins for 24 hours **(B)**, migration **(C)** and capillary tube formation **(D)**. Data are presented as mean ± SD in each experiment; n = 4. *: *p* < 0.05 compared with control.

In the *in vitro* study, early EPCs cultured from statin-naïve high-risk patients were treated with pitavastatin (10^−7^ M) or atorvastatin (10^−7^ M) for 24 hours. The adhesive function of early EPCs was significant enhanced by pitvastatin (control v.s. atorvastatin v.s. pitavastatin, 47.7 ± 14.0 vs. 51.1 ± 21.0 vs. 61.6 ± 27.8 cells/HPF, p = 0.04, n = 12) (Figure 3A). In proliferation assay, late EPCs were treated with pitavastatin or atorvastatin in various concentrations for 24 hours. The proliferation of late EPC was significantly inhibited by pitavastain with concentrations higher than 10^−6^ M, which was not seen in atorvastatin group (Figure 3B). Then, we chose the concentrations of 10^−7^ M of both statins in the *in vitro* studies. In migration assay, late EPCs treated with pitavastatin but not atorvastatin for 24 hours showed significant enhancement of migration ability (136.7 ± 14.7 v.s. 91.0 ± 20.7 number/HPF, p < 0.05) (Figure 3C). Moreover, in the *in vitro* angiogenesis assay, pitavastatin (10^−7^ M) but not atorvastatin significantly increased capillary-like patterns in high-power fields as compared with controls (161.5 ± 29.5 v.s. 123.1 ± 25.1% of control, p = 0.032) (Figure 3D).

**Figure 4:**
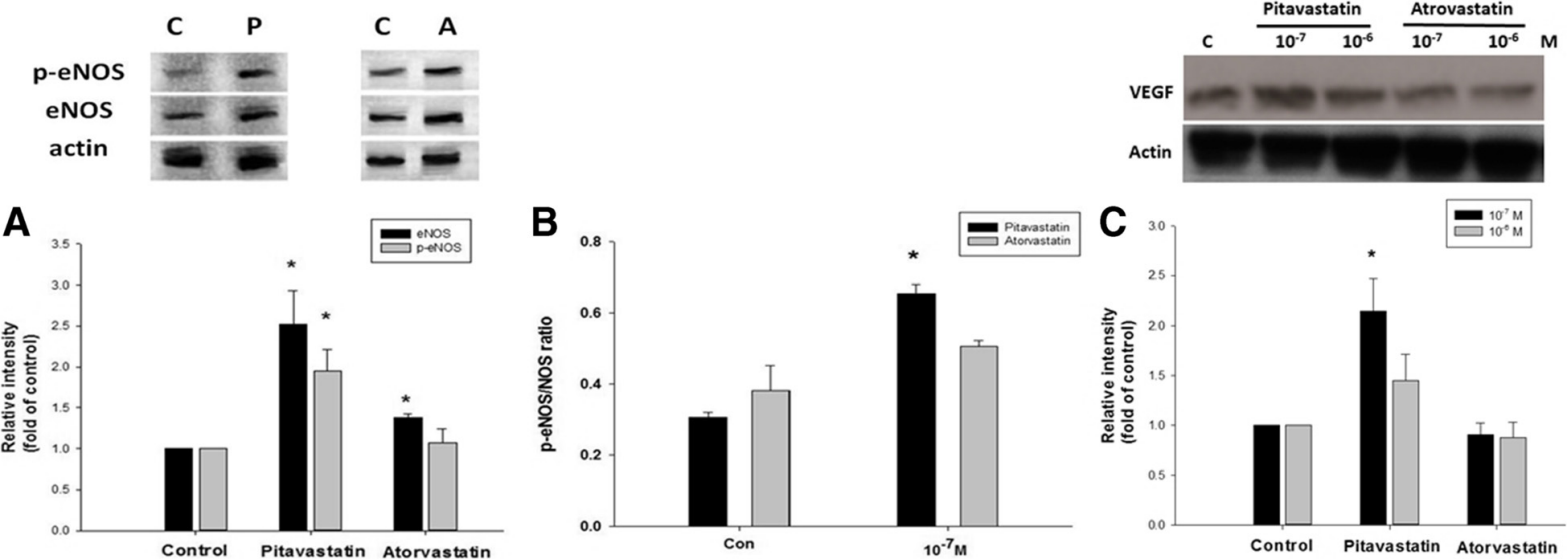
**Pitavastatin increased the endothelial nitric oxide synthase (eNOS) and vascular endothelial growth factor (VEGF) expression in human endothelial progenitor cells (EPCs).** Late EPCs were pretreated with pitavastatin and atorvastatin (10^−7^ M) for 24 hours. Both of pitavastatin and atorvastatin increased significantly total form of eNOS activation analyzed by Western blotting **(A)**. Ratio of phosphorylated eNOS over total eNOS significantly increased only in late EPCs after co-incubated with pitavasatin **(B)**. Pitavastatin (10^−7^ M) increased significantly expression of VEGF in early EPCs analyzed by Western blotting **(C)**. *: *P* < 0.05 was considered significant.

Finally, to elucidate the potential mechanisms of the effects of statins, we examined the protein expression of total form and phosphorylated eNOS in late EPCs after co-incubated with pitavastatin or atorvastatin (10^−7^ M) for 24 hours. Both statins significantly enhanced total form eNOS protein expressions (Figure 4A). However, only pitavastatin increased the ratio of phosphorylated eNOS over total eNOS expression (0.65 ± 0.03 vs. 0.51 ± 0.02, p < 0.05, n = 6) (Figure 4B), suggesting the direct effects of pitavastatin on EPC eNOS activation. Futhermore, only pitavastatin (10^−7^ M) increased the VEGF expression (2.14 ± 0.33 vs. 0.90 ± 0.11, p < 0.05, n = 6) (Figure 4C), suggesting the direct effects of pitavastatin on VEGF expression in early EPCs.

## Discussion

The cardinal findings of our study included that (1) while the treatment of pitavastatin or atorvastatin in an ordinary dose for 12 weeks significantly reduced serum total cholesterol, LDL-c and apolipoprotein B to a similar extent, only pitavastatin significantly increased serum VEGF levels and the number of circulating EPCs in high risk patients; (2) pitavasatin directly enhanced *in vitro* adhesion, migration, and tube formation with increased eNOS phosphorylation and VEGF expression in early or late EPCs from the patients; (3) atorvastatin treatment significantly reduced serum ADMA levels, a nature inhibitor of eNOS. Atorvastatin indirectly increased *in vitro* eNOS expression without altering eNOS phosphorylation and the function of EPCs. Taken the above together, long-term treatment with an ordinary dose of pitavastatin may increase circulating EPC number, which might be partially related to its direct effects on eNOS activation in EPCs and indirect effects by elevation of systemic VEGF. On the other hand, atorvastatin treatment did not increase circulating EPC number though did reduce serum ADMA levels. Further, atorvastatin did not alter eNOS expression and *in vitro* EPC function. Accordingly, in terms of chronic treatment with an ordinary dose, different statins such as pitavastatin and atorvastatin may have different effects on circulating EPCs. Our findings suggest that the vascular protection indicated by EPCs may vary with different statins, which might be not a class effect of statins.

It was previously shown that short-term treatment (less than 4 ~ 8 weeks) of some statins in a high dose could promote EPC mobilization from the bone marrow and ameliorate EPCs’ function, leading to vascular protection in different clinical conditions [[12]-[15],[17],[20]]. In the current study, our findings provide additional clinical evidence for the beneficial effects of long-term (more than 10 weeks) pitavastatin treatment in an ordinary dose on EPCs. The beneficial effects may be selective with pitavastatin and independent to lipid lowering since the reduction of serum cholesterol level was similar by both statins. Our findings are partially in line with the previous findings that short-term treatment of simvastatin (10 mg/day for 4 weeks) but not ezetimibe increased the number of EPCs in 10 patients with chronic heart failure [[17]]. Taken together, though the mechanisms have not been completely clarified, either pitavastatin or simvastatin may have the pleiotropic beneficial effects particularlly on circulating EPCs in different clinical settings.

**Figure 5:**
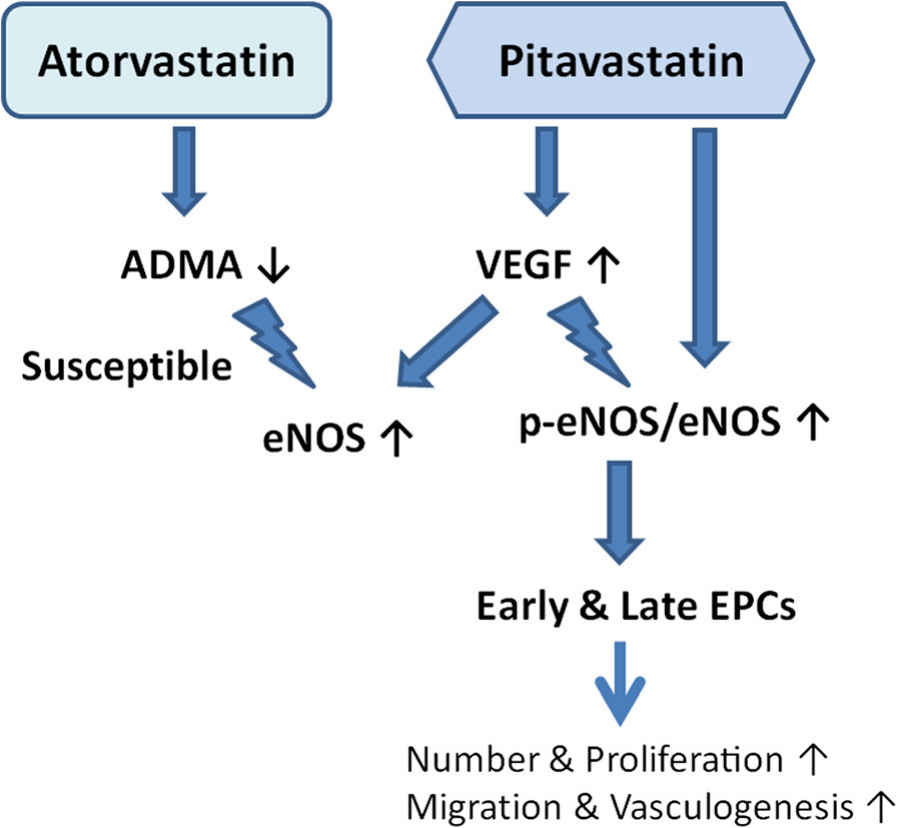
**Schematic representation of the possible mechanism of effects of pitavastatin and atorvastatin on endothelial progenitor cells (EPCs).** Atorvastatin decreased serum asymmetric dimethylarginine (ADMA) and pitavastatin increased serum vascular endothelial growth factor (VEGF), both increasing the expression of total form endothelial nitric oxide synthase (eNOS) in late EPCs. Only pitavastatin could increase circulating EPCs number and function via enhancing eNOS activity.

Several putative mechanisms of statin-induced EPCs mobilization were illustrated via increased endothelial NO availability, serum VEGF, receptor activator of NF-kappaB ligand (RANKL) and activation of matrix metalloproteinase (MMP)-2 and −9 [[21]-[25]]. The increase in MMP activity could result in degradation of the extracellular matrix and reduce cellular attachment within the bone marrow niches, leading to the mobilization of EPCs into circulation. In addition, HDL-c is also suggested as an determinant of endothelial function and associated with EPCs number in hypercholesterolemia patients [[26]]. Pitavastatin was considered as a statin that could significantly increase HDL-c in patients with metabolic syndrome or type 2 diabetes mellitus [[27]]. However, there was no significant effect of the changes of serum HDL-c on the changes of circulating EPCs by pitavastatin in our study. Whereas, it was previously shown that pitavastatin treatment for 4 weeks did not alter the EPC promoting efficacy in the chronic smokers. Thus, it is probable that short-term treatment with pitavastatin was not long enough to restore the circulating EPCs count [[28]]. However, in the present study, both serum VEGF level and the number of circulating EPCs were significantly increased after 12-week treatment of pitavastatin but not atorvasatin. In previous animal study, pitavastatin had been shown to increase serum VEGF levels in murine with ischemic limb [[29]]. Moreover, VEGF is a known EPC mitogen, which increased the proliferation rate of EPCs as well as the activities. After EPCs mobilization from bone marrow, reduced VEGF levels may result in the slow growth and suppressed the activation of circulating EPCs. In vitro cellular studies and animal results suggested that VEGF gene transfer would augment EPC proliferation, adhesion, incorporation into endothelial cell monolayers, and in vivo neovascularization [[30]]. In the contrast, recent report shows atorvastatin in ordinary dose (10 mg per day) would decrease serum concentration of VEGF in patients with type 2 diabetes [[31]]. In addition, direct effects of pitavastatin on EPC eNOS activation were found in our in vitro study. The key role of eNOS phosphorylation for a statin in promoting ischemia-induced angiogenesis had been demonstrated previously without accelerating tumor-associated angiogenesis [[32],[33]]. Taken together, it seems that beneficial effects of pitavastatin on circulating EPCs might be related to the increase in serum VEGF and the direct effects on eNOS activation (Figure 5).

In the present study, pitavastatin treatment mainly increased the number of CD34^+^KDR^+^ EPCs. The definition of EPCs, as the biomarkers for cardiovascular disease, remains an unresolved question [[34],[35]]. The scientific foundation of EPCs is based on the use of isolated hematopoietic CD34^+^ cells that were shown to give rise to endothelial marker expressing cells [[8]]. Because CD34 is also expressed on matured endothelial cells, the more immature marker CD133 has been used to select for putative EPCs [[36]]. Based on the definition of EPCs, the minimal antigenic profile should include at least 1 marker of stemness/immaturity (usually CD34 and/or CD133), plus at least 1 marker of endothelial commitment (usually KDR). Circulating EPCs have been defined as CD34^+^KDR^+^ by several investigators, as it was confirmed that this phenotype rather than others identifies cells capable of stimulating angiogenesis *in vivo* [[37]]. It was recently reported that CD34^+^KDR^+^ cells showed better relationships with CHD and response to statin therapy if restricted to the diminished CD45 gate [[38]]. Indeed, though there was no control group in this study, the number (0.021 ± 0.015%) of circulating CD34^+^KDR^+^ EPCs of our patients was much lower than that (1.2 ± 1.0%) of the local healthy subjects in similar age, and higher than that (0.007 ± 0.007%) in another group of age-matched patients with severe coronary artery disease in our previous study [[39],[40]]. Accordingly, the CD34^+^KDR^+^ EPCs may be the most representative one in all subsets of circulating EPCs in the present study.

Serum ADMA, as a natural eNOS inhibitor, has been linked to the risk for atherosclerosis. In the current study, atorvastatin treatment significantly decreased serum ADMA level. It is in line with the previous findings that atorvastatin treatment reduced serum ADMA levels in patients with ischemic stroke [[41]]. However, in our study, the effects on serum ADMA level were not translated to the beneficial effects on circulating EPCs. Indeed, in previous studies, while improving endothelial function and clinical outcomes, atorvastatin or simvastatin treatment did not alter plasma ADMA levels [[42],[43]]. Accordingly, the clinical beneficial effects of statins may be not related to the alteration of serum ADMA. Future study should clarify the exact role of ADMA in the modification of EPCs.

There are some limitations of the study. Given the limited patient number and limited study duration, we can not exclude the possibility that atorvastatin treatmet may improve EPCs with a larger dose for a longer period of duration. Besides, there were predominantly patients with T2DM and there were no clinical outcomes shown in the study. Thus, the current findings should be viewed for concept proofing rather than conclusive. Future study is required to determine if the beneficial effects of statin on circulating EPCs could be translated to the benefit in some specific clinical outcomes in the general cohort.

In conclusion, while both similarly reduced plasma lipids, pitavastatin treatment in an ordinary dose, rather than atorvastatin treatment, increased serum VFGF level and circulating CD34^+^KDR^+^ EPCs number. Besides, pitavastatin but not atorvastatin activated eNOS and improve *in vitro* function of EPCs. Though still waiting for further confirmation, our findings did support the concept that vascular pleiotropic protection effects could vary with different statins, which might not be a class effect in clinical settings. Given the increasing importance of circulating EPCs for vascular repair in patients with severe atherosclerosis and T2DM, our fiindings may also provide a potential rationale for clinical statin treatment in some particular conditions where the improvement of circulating EPCs should be taken into consideration.

## Abbreviations

EPCs: Endothelial progenitor cells

CHD: Coronary heart disease

LDL-c: Low density lipoprotein cholesterol

NOx: Total nitric oxide

ADMA: Asymmetric dimethylarginine

KDR: Kinase insert domain receptor

MTT: 3-(4,5-dimethylthiazol-2-yl)-2,5-diphenyltetrazolium bromide

eNOS: Endothelial nitric oxide synthase

SDF-1α: stromal cell-derived factor-1α

VEGF: Vascular endothelial growth factor

## Competing interests

The authors declared that they have no competing interests.

## Authors’ contributions

The study concept and design was done by LYL, CCH, SJL and JWC. TCW, PHH, HBL recruited the subjects and collected the clinical data. JSC and TTC performed the laboratory assays. Analysis and interpretation of data and drafting of the paper was done by LYL and JWC. All authored approved the final version of the paper.
